# Diagnosis of Portal Hypertension

**DOI:** 10.3390/diagnostics15212774

**Published:** 2025-10-31

**Authors:** Søren Møller, Karen V. Danielsen, Lise Hobolth, Christian Mortensen, Nina Kimer

**Affiliations:** 1 Department of Clinical Physiology and Nuclear Medicine, Center for Functional and Diagnostic Imaging and Research, Copenhagen University Hospital Hvidovre, DK-2650 Hvidovre, Denmark; karen.vagner.danielsen.02@regionh.dk; 2 Department of Clinical Medicine, Faculty of Health Sciences, University of Copenhagen, DK-2200 København, Denmark; nina.kimer@regionh.dk; 3 Gastro Unit, Medical Division, Copenhagen University Hospital Hvidovre, DK-2650 Hvidovre, Denmark; lise.hobolth@regionh.dk (L.H.); christian.otto.mortensen@regionh.dk (C.M.)

**Keywords:** cirrhosis, hepatic venous pressure gradient, measurement of portal pressure, non-invasive tests, alcoholic liver disease, metabolic dysfunction-associated steatotic liver disease

## Abstract

Chronic liver disease (CLD) imposes a major global health burden, with portal hypertension (PH) and its complications driven by complex pathophysiological mechanisms. Understanding these processes is essential for effective therapy. The hepatic venous pressure gradient (HVPG) is the gold standard for assessing portal hypertension, providing key diagnostic, prognostic, and therapeutic guidance—particularly in distinguishing its type and monitoring response to treatments such as non-selective beta-blockers. While non-invasive tests like elastography and serum biomarkers are valuable for screening and follow-up, they cannot fully replace HVPG when precise measurement is needed. HVPG contains not only prognostic information but also helps to decide if pharmacological therapy is indicated and to monitor therapeutic effects with reductions correlating with improved outcomes. In this review, we highlight the comprehensive management of patients with PH and the indications for measurement of HVPG.

## 1. Introduction

The burden of chronic liver disease (CLD) is increasing worldwide, with a prevalence of cirrhosis now averaging approximately 3% [1]. Cirrhosis is among the leading causes of mortality, morbidity and quality of life impairment [2,3]. The most common causes of cirrhosis include alcohol-associated liver disease (ALD), metabolic dysfunction-associated steatotic liver disease (MASLD), and hepatitis B and C virus-induced cirrhosis [2,4]. In particular, the prevalence of MASLD has increased because of increased rates of obesity, type 2 diabetes, and metabolic syndrome [5,6]. The clinical signs of cirrhosis vary widely, from asymptomatic hepatomegaly to jaundice, ascites, and hepatic encephalopathy.

Progression of liver disease, characterized by increasing fibrosis and steatosis, leads to a rise in portal pressure. Portal hypertension (PH) is a common and severe complication of CLD and is responsible for the classical complications of cirrhosis, such as bleeding from gastroesophageal varices, ascites, hepatorenal syndrome (HRS), hepatic encephalopathy, and disturbances of liver metabolism. Early identification and management of these complications are vital to improve patient outcomes and survival [7].

The risk of decompensation occurs when the hepatic venous pressure gradient (HVPG) exceeds 10 mmHg, which is also defined as clinically significant portal hypertension (CSPH) [8,9]. PH can be accurately diagnosed by measuring the difference between the pressure between the portal vein and that of the inferior caval vein, which represents the hepatic perfusion pressure. For practical purposes, HVPG is measured as the pressure difference between the wedged hepatic (WHVP) and free hepatic venous pressures (FHVPs). The level of CSPH independently predicts the development of hepatic decompensation, liver-related complications, hepatocellular carcinoma (HCC), and mortality [10]. An increase in HVPG above 10 mmHg is associated with a 6-fold increase in the risk of developing HCC [11,12].

To assess the presence and severity of portal hypertension, both invasive and non-invasive diagnostic techniques are utilized, including assessment of HVPG and elastography, respectively. An accurate diagnosis of PH is essential for effective identification of patients at risk and appropriate intervention. This narrative review is based on the most recent literature in the field and sets the stage for a comprehensive evaluation of PH and its complex relationship with cirrhosis.

## 2. Pathophysiology of Portal Hypertension

**Figure 1:**
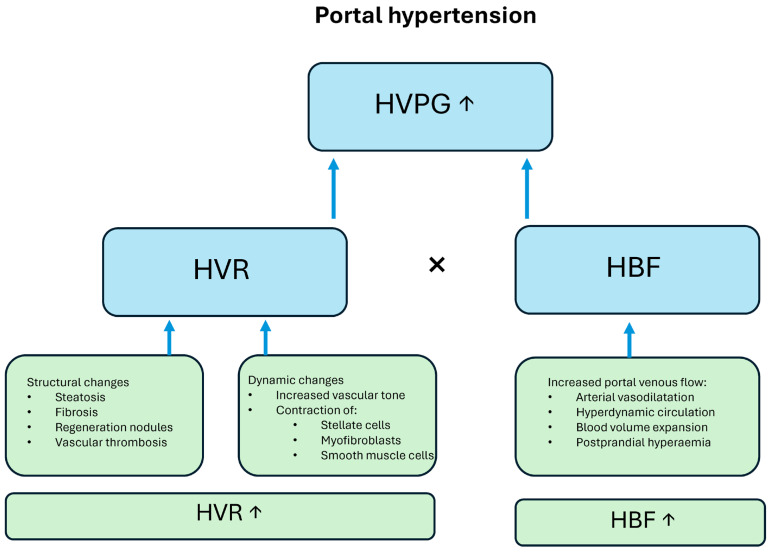
According to the law of Ohm the hepatic venous pressure gradient (HVPG) equals the hepatic vascular resistance (HVR) multiplied by the hepatic blood flow (HBF). Increased HVR is due to structural and dynamic changes and HBF increases because of hemodynamic changes.

At its core, portal hypertension arises from increased resistance to portal blood flow through the liver and increased portal venous inflow. According to Ohm’s law applied to the vascular system (ΔP = Q × R), the portal pressure (ΔP) is the product of portal blood flow (Q) and intrahepatic resistance (R). Therefore, both an increase in portal venous inflow and in the hepatic vascular resistance (HVR) contribute to the elevated portal pressure. HVR is composed of both static components, such as fibrosis, steatosis, and nodular regeneration in cirrhosis, and dynamic components, including active vasoconstriction mediated by hepatic stellate cell activation [13,14]. The structural or static lesions—steatosis (fat accumulation), fibrosis (scar tissue formation), and cirrhosis (nodular liver remodeling and architectural distortion)—mechanically obstruct hepatic sinusoids, increasing resistance to flow. The distribution and type of these static lesions depend on the etiology of the liver disease, which may differ between alcohol-related, metabolic-related, viral-related, or autoimmune liver diseases [15]. Therefore, the increase in portal pressure also depends on the presence and distribution of the static and functional changes. The pathophysiologic players of portal hypertension are summarized in Figure 1.

Among the functional players are a variety of cells, microvascular changes, and involvement of numerous vasoactive substances and activation of neurovascular systems [14]. An enhanced release or reduced clearance of endogenous vasodilators, notably nitric oxide, carbon monoxide, and endocannabinoids precipitates an arterial vasodilatation within the splanchnic circulation [16,17]. The dynamic component encompasses functional players at the cellular and vascular levels. Sinusoidal endothelial cells (SECs) produce NO, endothelins (ETs), prostanoids, and prostaglandins that act on hepatic stellate cells (HSCs), which possess receptors for ET-1, angiotensin-2 (AT-II), tissue inhibitor of metalloproteinase-1 (TIMP-1), and thrombin [18,19]. HSCs surround the sinusoids in the space of Disse, and through their contractile properties, they contribute to regulating the sinusoidal blood flow [14].

This vasodilatory response is a cornerstone of the compensatory mechanisms seen in portal hypertension. The resulting hyperdynamic circulation is characterized by a high cardiac output alongside a paradoxical drop in arterial blood pressure and central and arterial blood volume, which further stimulates compensatory mechanisms such as the renin-angiotensin-aldosterone system (RAAS) and sympathetic nervous stimulation, perpetuating splanchnic vasodilation and increasing splanchnic blood flow [14,20]. This increased splanchnic inflow, combined with high HVR, results in elevated HVPG. As the disease progresses, collateral circulation develops to divert blood around the high-resistance liver, which may manifest clinically as varices, ascites, and other complications of decompensated portal hypertension [16].

Mechanobiology is a rapidly growing field at the intersection of biology, physics, and engineering. In the context of portal hypertension, mechanobiological processes play crucial roles in liver fibrosis, vascular remodeling, and overall disease progression [21]. Briefly, mechanosensing detects increased portal pressure through HSCs and liver sinusoidal cells and converts forces into biochemical signals by mechanotransduction that elicits a mechanoresponse in PH, resulting in fibrosis, angiogenesis, and increased HVR [22].

Understanding the hemodynamic and molecular basis of portal hypertension is essential for targeted therapeutic interventions, which aim to reduce both the resistance within the liver and the excessive splanchnic blood flow contributing to disease progression [23].

## 3. Complications of Portal Hypertension

As the changes in the liver progress, portal pressure increases with the formation of portosystemic collaterals and development of the decompensated stage with associated complications such as ascites, bleeding esophageal varices, encephalopathy, and dysfunction of the kidneys, heart, and lungs [24,25].

Portal hypertensive splanchnic vasodilation and activation of renal sodium retention drive progressive fluid accumulation as ascites and edema [26]. Approximately 60% of patients with cirrhosis develop ascites within ten years, and the two-year mortality after ascites reaches about 50% without liver transplantation [27].

Portal hypertension forces blood to form portosystemic collaterals, leading to gastroesophageal varices. Esophageal varices appear when HVPG exceeds ~10 mmHg defined as CSPH [8]. These thin-walled submucosal veins are prone to rupture, and acute variceal hemorrhage occurs in 5–15% of patients per year with a high morbidity and mortality averaging more than a 20% six-week mortality despite therapy [28]. Accordingly, screening endoscopy and prophylactic treatments with non-selective beta-blockers, endoscopic ligation, or shunting procedures such as insertion of a transjugular-intrahepatic portosystemic shunt (TIPS) are standard care to prevent life-threatening variceal bleeding [8].

Hepatic encephalopathy (HE) results from neurologic dysfunction due to liver failure and portosystemic shunting of toxins. HE presents as a spectrum from subtle cognitive impairment to confusion, asterixis, and coma, and is graded by mental status according to the West–Haven criteria [29]. It significantly worsens patient prognosis and is often precipitated by GI bleeding, infection, or metabolic disturbances.

HRS is a form of functional renal failure in advanced cirrhosis with portal hypertension. Clinically, HRS manifests with progressive oliguria, rising creatinine and hyponatremia, and it carries a grave prognosis—type HRS-AKI with a rapid onset often leads to death within weeks without a liver transplantation [29,30].

## 4. Measurement of the Portal Pressure

**Figure 2:**
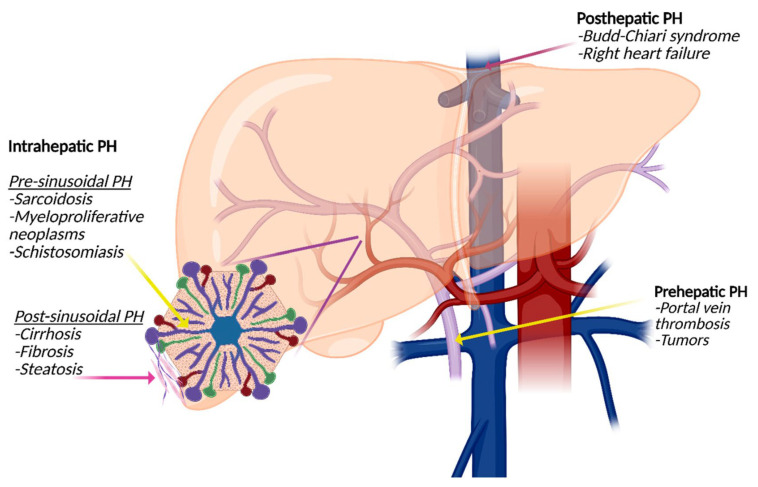
Types of portal hypertension (PH): Prehepatic PH is often caused by portal vein thrombosis or tumor-related thrombosis. Intrahepatic PH is divided into presinusoidal PH, for example, caused by sarcoidosis or schistosomiasis, and post-sinusoidal PH caused by cirrhosis of all causes. Posthepatic PH is most often associated with the Budd–Chiari syndrome or right-sided heart failure.

According to the anatomical location of the lesion, PH can be categorized as prehepatic, intrahepatic, or post-hepatic (Figure 2). The most common cause of prehepatic PH is portal vein thrombosis. Intrahepatic PH can further be divided into pre- and post-sinusoidal PH. Alcohol-related cirrhosis is most often associated with post-sinusoidal PH, whereas inflammatory and malignant conditions such as schistosomiasis can result in a pre-sinusoidal PH. Post-hepatic PH is caused by venous outflow obstruction with hepatic vein thrombosis, also known as the Budd–Chiari syndrome, right-sided heart failure, and cancer as leading causes.

The major indications for measuring HVPG are (1) to reveal the type of PH relating to the specific lesion as outlined above; (2) to distinguish between other causes of fluid retention such as for example right ventricular heart failure; (3) to measure the degree of PH before initiation treatment with non-selective betablockers and response to betablockade; (4) to achieve prognostic information relating to risk of esophageal bleeding, decompensation, development of HCC, and mortality; (5) to monitor effects of medical treatment; and (6) to measure portal pressure prior to TIPS insertion, surgery, and liver transplantation (see Table 1).

### 4.1. Technical Aspects

The gold standard of assessing PH is the minimally invasive measurement of the HVPG calculated as the difference between WHVP and FHVP. WHVP reflects sinusoidal pressure when the catheter is wedged in a hepatic vein, while FHVP reflects systemic venous pressure. The normal HVPG ranges from 1 to 5 mmHg. A value ≥6 mmHg indicates portal hypertension, while values ≥10 mmHg are considered CSPH and are predictive of complications. Levels of HVPG > 12 mmHg are considered severe PH.

The liver vein catheterization involves puncture typically via the internal jugular, femoral, or brachial vein. In the supine position the vein is punctured under ultrasound-guided local anesthesia. Sedation is usually not needed. A venous introducer is inserted in the vein, and during fluoroscopic control, a Swan-Ganz balloon 7F catheter is advanced to a hepatic vein. The position can be documented by use of diluted contrast dye. Pressures are electronically obtained by a capacitance transducer calibrated to a baseline pressure of 0 mmHg with the midaxillary line being the zero pressure level [31]. The scale range should be adjusted to 50 mmHg, whereafter the FHVP and WHVP are measured over at least a period of 20 and 60 s, respectively, or until stabilization. WHVP is measured after inflation of the balloon with air sufficiently to occlude the vein and avoid artifacts. Contrast dyes can be used to reveal eventual intrahepatic venous communications. All pressures should be measured at least three times and average values applied. Successive measurements should be within a variation of 2 mmHg; otherwise, the technique and/or the position should be reevaluated. Before finalizing the pressure measurements, the FHVP should be measured approximately 2 cm from the hepatic vein outlet as the pressure in the proximal FHVP. Similarly, the pressures should be measured in the inferior caval vein and in the right atrium.

Pitfalls in the HVPG measurements include overestimation of the FHVP and underestimation of the WHVP. An increased FHVP suggests obstruction of the inferior caval vein or cardiac failure. Clotting of the catheters is another pitfall, which can be omitted by frequent flushing with saline. Underestimation of the WHVP is seen in the case of partial balloon occlusion and in the presence of intrahepatic communications as for example seen in MASLD. Complications to liver vein catheterization are seldom and include local bleeding at the puncture site, hematoma, and arrythmias during the passage of the right atrium via the transjugular route. The procedure should be avoided in case of a history of allergy to iodinated contrast media, severe thrombocytopenia (platelet levels < 20 × 10^−9^) or a very prolonged international normalized ratio (>2.5). Table 2 shows a practice checklist for the measurement of HVPG.

**Figure 3:**
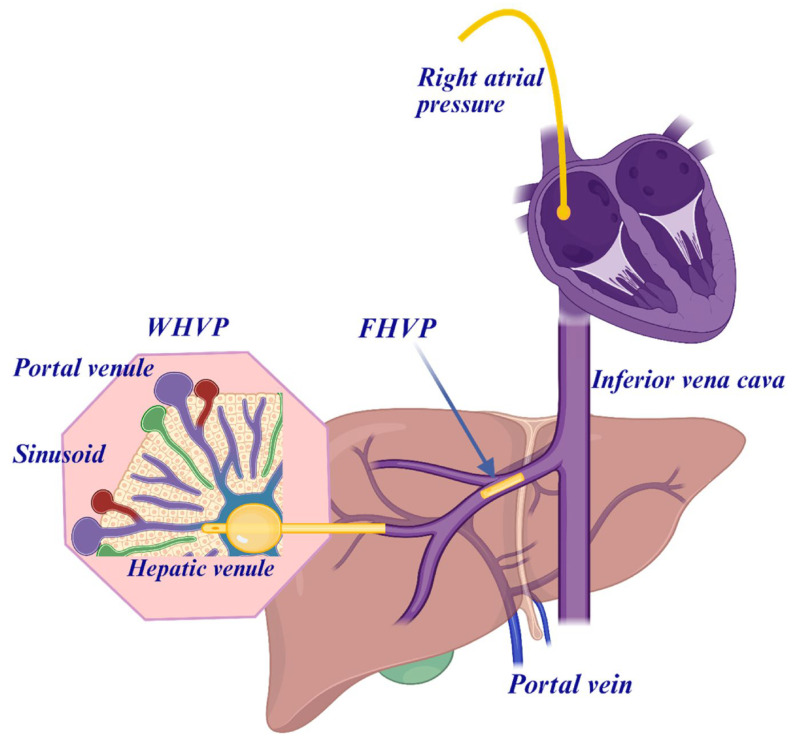
The hepatic venous pressure gradient (HVPG) can be measured by the femoral venous or the transjugular venous route during a liver vein catheterization. The hepatic venous pressure gradient (HVPG) is measured as the difference between the wedged hepatic venous pressure (WHVP) and the free hepatic venous pressure (FHVP). WHVP is achieved by a balloon occlusion with, for example, a Swan-Ganz catheter approximately measuring the portal venous pressure.

Using the transjugular route, the procedure can be accompanied by a transjugular liver biopsy, which is also performed under fluoroscopic control with contrast media prior to and after the biopsy (Figure 3).

### 4.2. Interpretation of Portal Pressure Measurements

**Figure 4:**
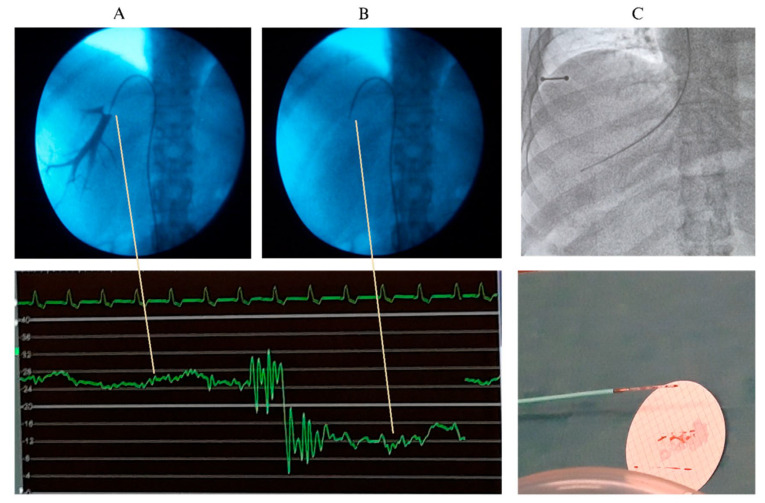
A liver vein catheterization through the femoral route with measurement of the wedged hepatic venous pressure (WHVP) with balloon occlusion and use of contrast dye (**A**) and the free hepatic venous pressure (**B**). Below the pressure measurement, where the hepatic venous pressure gradient (HVPG) is calculated as the difference between WHVP (high level curve) and the FHVP (low level curve). Panel (**C**) shows a transjugular biopsy.

Figure 4 shows a typical tracing of HVPG in a cirrhotic patient with post-sinusoidal PH with well-defined pressure measurements of FHVP and WHVP. In case of prehepatic PH, the presence of a portal vein thrombosis complicates the interpretation of the portal pressure measurements. HVPG may be measured as normal or falsely low because it reflects the sinusoidal pressure, while the thrombosis causes pre-hepatic resistance. In these patients, Doppler ultrasound examination and CT/MRI are essential for the final diagnosis. However, HVPG may still be helpful in evaluating coexisting cirrhosis or HVR. In non-cirrhotic PH conditions like idiopathic PH, nodular regenerative hyperplasia, and congenital hepatic fibrosis can present with PH without cirrhosis. In non-cirrhotic PH, WHVP, and FHVP may typically be normal in presinusoidal cases, wherefore HVPG may not reflect the clinical severity and has limited utility in purely presinusoidal diseases.

A particular challenge in accurate portal pressure measurements exists in MASLD patients in whom the HVPG begins to rise in early stages and fibrosis per se is not a prerequisite for the development of PH. However, there seems to be a strong correlation between HVPG and stage of fibrosis in MASLD [32]. The HVR and portal pressure begin to increase because of reduced sinusoidal space owing to the increasing amount of lipid, fibrotic tissue, and cirrhotic nodules. However, it is generally accepted that the measured portal venous pressure is underestimated in MASLD. This may partly be due to the presence of intrahepaticsinusoidal communications, most pronounced in steatosis and in milder degrees of fibrosis, but may disappear in the cirrhotic stage because of obstruction by the cirrhotic scar tissue [33]. Decompensation in MASLD patients seems to appear at HVPG levels of approximately 4 mmHg lower than expected in patients with other etiologies of cirrhosis, such as in alcohol-induced liver disease. Therefore, HVPG determination has a lower accuracy, which explains why the real HVPG is underestimated, which should be taken into consideration in the interpretation of the severity of PH in MASLD patients [34].

## 5. Non-Invasive Tests for Portal Hypertension

As mentioned above, measurement of the HVPG by a liver vein catheterization represents the gold standard method to assess type and severity of PH. However, several non-invasive surrogate markers of PH have been proposed, and since they are more applicable and suitable for continuous monitoring and repeated measurements, these are often more commonly used. Although some non-invasive measures may exclude CSPH, they have so far been unable to differentiate between degrees of fibrosis, although they may be accurate in separating early from advanced fibrosis. Non-invasive methods to assess PH rely on physical or biologic methods. Physical methods assess organ stiffness by ultrasound or MRI-techniques, whereas the biological approach utilizes quantification of several serum biomarkers that have been associated with the stage of fibrosis. In addition, non-invasive scoring systems and hematologic parameters have been applied. The most often used non-invasive tests are summarized in Table 3.

### 5.1. Ultrasound and Elastography

Conventional ultrasound can evaluate the size of the spleen, detect portosystemic collaterals, and assess signs of PH such as splenomegaly and enlarged portal vein. Transient elastography (TE) has been used for more than 20 years to evaluate liver stiffness by measuring the velocity of shear waves propagating through liver tissue. It is a technique sensitive to tissue stiffness and measures the changed elasticity resulting from the increased amount of fibrosis. Hence, TE (FibroScan) measures liver stiffness as a surrogate marker for fibrosis, which correlates with portal hypertension [15,35]. Elevated liver stiffness measurements suggest increased portal pressure [36]. In patients with ALD there is evidence of a correlation between a fibroscan and measurement of HVPG. Thus, TE values below 10 kPa rule out CSPH; values between 10 and 15 kPa are suggestive of CSPH, and values above 15 kPa are highly suggestive of CSPH [8]. TE values do not quantify the severity of PH, but correlate closely with HVPG values up to 10 mmHg; however, with a liver stiffness value above 13.6 kPa, the correlation is less accurate [37]. When HVPG values exceed 10–12 mmHg, the portal pressure becomes largely independent of fibrosis and the characteristic hemodynamic changes of severe PH are no longer reflected by the liver stiffness measurement. Taken together, TE is an imperfect surrogate marker for HVPG but adds valuable information on the stage of fibrosis.

Similarly, spleen stiffness can quantitatively be assessed using elastography techniques. Increased spleen stiffness correlates with higher portal pressures and the presence of esophageal varices [35].

### 5.2. Magnetic Resonance Imaging

MR Elastography (MRE) offers the ability for a non-invasive assessment of liver and spleen stiffness with high accuracy and can visualize collateral circulation and varices. Both liver and spleen stiffness correlate with the degree of PH [38]. Moreover, it provides comprehensive anatomical and functional information relevant to PH [39]. In addition, phase-contrast MRI can quantify portal flow and, when combined with collateral mapping, reaches a diagnostic accuracy on par with elastography [40]. However, the modality is still expensive and time-consuming compared to ultrasound elastography, and it still needs to be validated in larger cohorts.

### 5.3. Biomarkers and Scoring Systems

Many biomarkers have been validated for the diagnosis of fibrosis in CLD. Indirect scores with markers of liver function include the aspartate-to-platelet ratio index (APRI) and the fibrosis-4 score (FIB-4) [41,42]. The APRI score is calculated as (AST/upper limit of normal) × 100/platelet count) where FIB-4 combines age AST, ALT, and platelet count to estimate liver fibrosis severity and risk of PH [42]. Reduced platelet count is common in PH due to splenic sequestration and reduced thrombopoietin production. A low platelet count per se supports the suspicion of significant PH [42]. In addition, the platelet/spleen ratio improves the prediction of PH and esophageal varices [43]. Several studies have shown that FIB-4 and APRI can accurately differentiate significant fibrosis from nonfibrotic histology. However, their performance in predicting esophageal varices and PH has been discouraging [41]. The direct scores with assessment of serum extracellular matrix components and intermediates of fibrogenesis include the enhanced liver fibrosis (ELF) test and the FibroTest [44]. The ELF test measures serum concentrations of extracellular matrix turnover markers, reflecting fibrogenesis and fibrosis severity. The Fibro Test is a composite panel including alpha-2-macroglobulin, haptoglobin, apolipoprotein A1, GGT, and bilirubin, integrated into a score indicating liver fibrosis stage [45]. Their accuracy is reasonable to distinguish between the absence of or little fibrosis and advanced stages of fibrosis. Both the FibroTest and the ELF-test have shown different prognostic accuracies to predict outcomes in different patient populations of cirrhosis, and this may impact the clinical applicability of the tests [42,44].

Although invasive determination of PH during a liver vein catheterization remains the gold standard, non-invasive tests have proved to be fairly effective in assessing CSPH. In particular, imaging-based techniques appear to have a somewhat higher accuracy than blood-based tests [41]. Neverthesless, it should be considered that a significant correlation between a biomarker and HVPG does not state a pathophysiological relationship.

## 6. Prognostic Role of PH Measurements

HVPG both correlates with the presence of complications of PH and serves as a strong prognostic marker for various outcomes in cirrhosis, including mortality, ascites, variceal bleeding, HCC, HRS, and response to interventions such as transjugular intrahepatic portosystemic shunt (TIPS); see Table 4. In addition to quantifying the diagnosis and level of PH, HVPG also provides powerful prognostic information regarding, for example, the risk of ascites and HCC, effects of pressure-lowering treatment and survival.

*Mortality.* In compensated cirrhosis, HVPG ≥ 10 mmHg marks CSPH and predicts transition to decompensation and death. Patients with HVPG < 10 mmHg have a 90% chance of remaining free of decompensation at two years [46]. In decompensated cirrhosis, each 1 mmHg rise in HVPG independently increases mortality by approximately 3% [46]. An HVPG > 16 mmHg bears the highest risk of death, while thresholds ≥ 20 mmHg are linked to markedly reduced one-year survival independent of liver function scores like Model of End-stage Liver Disease (MELD) and Child–Pugh [47]. Reductions in HVPG of ≥10% from baseline or to <12 mmHg in response to pharmacologic therapy correlate with improved survival [8].

*Ascites.* Development of decompensation correlates with the increase in HVPG, and patients with ascites have higher HVPG than those without [47]. CSPH (HVPG ≥ 10 mmHg) identifies patients at risk for their first episode of ascites, while values ≥ 12 mmHg predict refractory ascites and HRS. Cirrhotic patients of different etiologies, including MASLD with HVPG < 10 mmHg, have a low risk of developing ascites and low liver-related mortality [10,11].

*Variceal bleeding.* The risk of bleeding from esophageal varices increases once HVPG exceeds 10 mmHg and the risk rises sharply with HVPG above 12 mmHg [8]. In acute variceal bleeding, HVPG ≥ 20 mmHg identifies patients at very high risk of early rebleeding and death [48,49]. A reduction in HVPG to <12 mmHg or by ≥20% from baseline, induced by non-selective beta-blockers or carvedilol, defines *hemodynamic responders*. These patients have an ~50% lower risk of first variceal bleeding and a >60% reduction in the risk of rebleeding [50,51,52]. During a liver vein catheterization with measurement of HVPG, an intravenous beta-blocker test can be performed with a fluid suspension of propranolol. The response can immediately be observed where an HVPG reduction >10% from baseline is the target to define response in primary prophylaxis [53]. The evaluation of the acute response to intravenous non-selective betablockers is useful and cost-effective to guide therapeutic decisions in cirrhotic patients with PH [54].

*HCC.* HVPG independently predicts de novo development of HCC. Patients with HVPG ≥ 10 mmHg have an annual HCC incidence of 2.1% versus 0.35% for HVPG < 10 mmHg [55]. Hence, PH is an independent predictor of HCC development and is associated with a 6-fold increase in risk of HCC [56].

*HRS.* Severe PH contributes to circulatory dysfunction, splanchnic vasodilation, and sodium retention, predisposing to HRS. An HVPG > 12 mmHg and higher values correlate with increased risk of HRS onset and mortality [57].

*TIPS.* Insertion of a TIPS aims to lower portal pressure in patients with refractory ascites or recurrent esophageal bleeding [58]. A post-TIPS HVPG < 12 mmHg or ≥20% decrease from pre-TIPS is associated with markedly lower rebleeding rates and better control of ascites and bacterial peritonitis, translating into improved survival [29]. However, depending on the size of the shunt, there is an increased risk of hepatic encephalopathy after the procedure [29,59].

## 7. Summary and Conclusions

CLD remains a major global health burden, causing significant morbidity, mortality, and healthcare use. Progression to PH leads to complications like variceal bleeding, ascites, HRS, hepatic encephalopathy, and HCC, driven by complex vascular changes in the liver. Understanding these mechanisms is the key to effective treatment.

HVPG measurement is the gold standard for assessing PH, providing accurate classification and guiding therapy, especially when the cause is unclear or precise monitoring is needed. Though highly accurate, HVPG is increasingly supplemented by non-invasive tools like TE, biomarkers, and imaging, which aid screening but cannot fully replace HVPG for treatment decisions.

HVPG >10 mmHg indicates clinically significant PH and predicts complications. Monitoring HVPG responses to therapies like non-selective beta-blockers helps improve outcomes, with a ≥20% reduction or decrease below 12 mmHg as therapeutic targets.

Future research should enhance non-invasive methods, identify biomarkers for personalized risk, and develop targeted treatments. Advances in omics, AI, and imaging promise more precise, patient-centered management of PH in CLD.

## Figures and Tables

**Table 1:** Indications for measurement of the portal pressure.

Characterization of PH	Increased HVPG owing to an increase in WHVP indicates an increase in sinusoidal pressure, which is most often due to cirrhosis and intrahepatic PH HVPG < 5 mmHg with a normal WHVP and FHVP is typical of presinusoidal portal hypertension Increased FHVP and WHVP are frequently due to post-hepatic PH
Differential diagnosis	Other conditions with fluid retention, e.g., heart failure
Assessment of the degree of PH	Before initiation of treatment with, for example, NSBB
Prognostic information	Risk of decompensation, bleeding from esophageal varices, development of HCC, and mortality
Monitoring direct effects of betablockers	- Direct NSBB response with HVPG measurement before and after i.v. NSBB. - Indication for primary prophylaxis (HVPG > 10 mmHg)
Prior to invasive procedures	- Before surgery, TIPS-insertion, liver transplantation - Evaluation after TIPS-insertion

PH: portal hypertension; HVPG: hepatic venous portal gradient; WHVP: wedged hepatic venous pressure; FHVP: free hepatic venous pressure; HCC: hepatocellular carcinoma; NSBB: non-selective betablockers; TIPS: transjugular intrahepatic portosystemic shunt.

**Table 2:** Practice checklist for measurement of the hepatic venous pressure gradient (HVPG).

*Before measurement* Basic equipment - Digital x-ray system - Pressure recorder and transducer system - Ultrasound system for real-time venous access guidance - Sterile utensils (needles, guidewires, sheaths, balloon-tipped catheters, syringes etc.) Choose the most appropriate route - Femoral vein - Brachial vein - Jugular vein (allows transjugular liver biopsies) Pressure measurement - Select scale range (0–40/0–50 mmHg) - Calibrate or use precalibrated transducers connected to a monitoring system - Place the transducer at the level of the patient’s mid-axillary line Patient preparation - Ensure patients comfort and security - Prepare ECG surveillance - Avoid deep sedation - Disinfect relevant skin area - Insert ultrasound guided vascular sheath - Insert through the sheath a balloon-tipped catheter - Advance the catheter under x-ray control to a stable position in the hepatic vein *During measurement* - Measure the free hepatic venous pressure (FHVP) 2–3 cm from the hepatic vein outlet for at least 30 s - Inflate the balloon of 10–12 mm to measure the wedged hepatic venous pressure (WHVP) for at least 60 s. - Repeat the measurement at least in triplicate and calculate HVPG. - Measure proximal FHVP 1–2 cm from the hepatic vein outlet - Measure the inferior caval vein pressure - Measure the left atrial pressure to exclude coexisting left cardiac failure *After measurement* - Remove the sheaths and apply manual compression as appropriate - Maintain supine rest (femoral access) or upleft position (jugular access) as appropriate - Discharge the same or the next day in uncomplicated procedures.

**Table 3:** Currently used non-invasive tests for clinically significant portal hypertension (CSPH).

Type of Non-Invasive Test	Interpretation and Cut-Offs
Vibration-controlled elastography/Fibroscan	<15 kPa + platelets > 150 G/L → CSPH ruled out; ≥25 kPa → CSPH very likely
Spleen stiffness measurement	≥40 kPa highly specific for CSPH; <21 kPa rules out CSPH
MR Elastrography (MRE)	MRE measures both hepatic and splenic stiffness; a liver MRE ≥ 5.7 kPa or spleen MRE ≥ 8 kPa points toward CSPH
Fibrotest (FIB-4)	Age × AST ÷ (Platelets × √ALT) > 3.25: advanced fibrosis, more likely CSPH
Aspartate-to-platelet ratio index (APRI)	AST ÷ ULN AST ×100 ÷ Platelets >1.0 suggesive of CSPH
Enhanced Liver Fibrosis (ELF)-test	HA + PIIINP + TIMP-1 (proprietary algorithm) > 11 predicts decomp events and CSPH
Fibro test	haptoglobin, ApoA1, GGT, TBil ± age/sex > 0.75 (F4) → high risk of CSPH
Platelet count	<150 G/L: suspect CSPH

AST: aminoaspartatetranspherase; ULN: upper limit of normal; HA: hyaluronic acid; PIIINP: pro-collagen 3 N-terminal peptide; TIMP-1: Tissue inhibitor of metalloproteinase-1; ApoA1: Apolioprotein A1; GGT: gamma glutamyl transpeptidase; TBil: total bilirubin.

**Table 4:** Prognostic information of portal pressure assessment with relevant hepatic venous pressure gradient (HVPG) thresholds.

Outcome	Critical HVPG Threshold	Prognostics Implication
Decompensation/Ascites	≥10 mmHg	Risk of ascites and decompensation
Variceal Formation/Bleeding	≥10/≥12 mmHg; ≥20 for bleeding	Bleeding risk onset; high mortality if ≥20 mmHg
Mortality	>16–17 mmHg	Higher 1- and 2-year mortality rates
HCC Risk	>10 mmHg	Independent increased risk of HCC development
Surgical/HRS Risk	>16–20 mmHg	High peri-operative and renal dysfunction risk
Post-TIPS Improvement	≤12 mmHg or ≥50% reduction	Better control of bleeding/ascites, improved survival

## Data Availability

No new data were created or analyzed in this study. Data sharing is not applicable to this article.

## Abbreviations

ALD	Alcoholic liver disease
APRI	Aspartate-to-platelet ratio index
ATII	Angiotensin-II
CLD	Chronic liver disease
CSPH	Clinically significant portal hypertension
FIB-4	Fibrosis-4 index
ELF	Enhanced liver fibrosis score
ET	Endothelin
FHVP	Free hepatic venous pressure
HCC	Hepatocellular carcinoma
HRS	Hepatorenal syndrome
HSC	Hepatic stellate cells
HVPG	Hepatic venous pressure gradient
HVR	Hepatic vascular resistance
MASLD	Metabolic dysfunction-associated steatotic liver disease
MELD	Model of End-Stage Liver Disease
MRE	Magnetic resonance Elastography
MRI	Magnetic Resonance Imaging
PH	Portal hypertension
SEC	Sinusoidal endothelial cell
TE	Transient elastography
TIMP-1	Tissue inhibitor of metalloproteinase-1
TIPS	Transjugular intrahepatic portosystemic shunt
WHVP	Wedged hepatic venous pressure
